# Modeling Asymmetry of a Current–Voltage Curve of a Novel MF-4SC/PTMSP Bilayer Membrane

**DOI:** 10.3390/membranes12010022

**Published:** 2021-12-24

**Authors:** Anatoly N. Filippov, Natalia A. Kononenko, Natalia V. Loza, Daria A. Petrova

**Affiliations:** 1 Department of Higher Mathematics, National University of Oil and Gas Gubkin University, 119991 Moscow, Russia; kononenk@chem.kubsu.ru (N.A.K.); petrova.msu@gmail.com (D.A.P.); 2 Department of Physical Chemistry, Kuban State University, 350040 Krasnodar, Russia; nata_loza@mail.ru; 3 Skolkovo Institute of Science and Technology, 121205 Moscow, Russia

**Keywords:** bilayer MF-4SC/PTMSP composite membrane, asymmetry of current–voltage curve, membrane sensors and diodes, “fine porous membrane” model

## Abstract

A novel bilayer cation-exchange membrane—consisting of a thick layer of a pristine perfluorinated membrane MF-4SC (Russian equivalent of Nafion^®^-117) and a thinner layer (1 μm) of the membrane, on a base of glassy polymer of internal microporosity poly(1-trimethylsilyl-1-propyne) (PTMSP)—was prepared and characterized. Using the physicochemical characteristics of one-layer membranes MF-4SC and PTMSP in 0.05 M HCl and NaCl solutions, the asymmetric current–voltage curves (CVC) of the bilayer composite were described with good accuracy up to the overlimiting regime, based on the “fine-porous membrane” model. The MF-4SC/PTMSP bilayer composite has a significant asymmetry of CVC that is promising for using it in electromembrane devices, such as membrane detectors, sensors, and diodes.

## 1. Introduction

The asymmetry of transport properties is an important feature of bilayer membranes. When the direction of the driving force (pressure or concentration gradients, electric field) changes, the flux density of solvent, solute [1,2], and electric current [2,3,4,5,6,7] can change significantly. In the latter case, an asymmetry of the current–voltage curves (CVC) is observed. It leads to a significant difference in the limiting current density when the orientation of the membrane in the measuring cell changes. The asymmetry of the CVC was also detected at single pores of the track-etched membrane [8]. This property can be promising for creating membrane diodes, when a bilayer membrane passes current well in one direction, and practically does not pass it in the contrary direction [9,10].

For the first time, the asymmetry of the current–voltage curve was observed, apparently, in lipid bilayer membranes [11,12,13]. Due to exposure of cells to strong pulsed electric fields, it is possible to regulate the conductivity of the bilayer cell membrane and change its state from non-porous to porous (electroporation) [14]. Regulation of the conductivity of planar lipid bilayer by means of soluble amyloid oligomers is extremely important in the treatment of Alzheimer’s disease [15]. To obtain bilayer materials based on ion-exchange membranes, a layer is applied to the substrate material, which may contain the fixed ions having the same [16] or opposite charge sign [2,17] as the fixed ions of the substrate have. In particular, the electrochemical properties of two-layer ion-exchange membranes, consisting of a thick heterogeneous membrane layer MC-40 and a thin homogeneous membrane layer MF-4SC, have been studied in [16], both theoretically and experimentally. The study of asymmetric membranes, whose surface is modified by multilayers containing polyelectrolytes of different polarities, which made it possible to give the membranes selectivity to single-charged ions, is presented in [18].

The aim of the present work is to establish and quantify the asymmetry of the current–voltage curve of a novel bilayer composite membrane, based on a thick layer (219 µm) of cation-exchange perfluorinated membrane MF-4SC and a thin (1 µm) non-conducting layer of a glassy polymer of internal microporosity poly(1-trimethylsilyl-1-propyne) (PTMSP), depending on the direction of the external electric field. Two factors determined our choice of the PTMSP polymer—(i) its physicochemical properties are close to those of the MF-4SC polymer (porosity/free volume, hydrophobicity, degree of swelling, both polymers are stable in acid and ethyl alcohol) and (ii) PTMSP is practically non-charged. It was recently shown that the ζ-potential of the PTMSP membrane surface at the 0.5 g/L NaCl concentration in the ethanol–water solutions changes almost linearly from −15 mV at 0% of ethanol content to 0 mV at 100% of ethanol in the mixture [19]. At the same time, we have theoretically shown that bilayer membranes with the highest difference in the modules of effective exchange capacities have the greatest asymmetry [1,3]. This means that one of the membrane layers must be neutral for reaching a maximum asymmetry. Therefore, the layered membrane composites with high asymmetry of current–voltage curves, such as MF-4SC/PTMSP, are important for potential applications, such as membrane sensors, detectors, and diodes [9,10].

## 2. Experimental

### 2.1. Membrane Casting

A thick layer of the composite membrane MF-4SC/PTMSP was cast from solution of the MF-4SC polymer in lithium form (7.2 wt% in dimethylformamide, 0.98 mmol/g exchange capacity, Plastpolymer, St. Petersburg, Russia). The MF-4SC polymer is a perfluorinated homogeneous-type membrane containing sulfonic acid groups. On the dried and cooled layer 1 of the MF-4SC, a cooled 2% solution of the PTMSP in CCl_4_ was applied with the addition of 0.3–1.0 mL of ethyl alcohol to form layer 2. Layer 2 was dried at room temperature for 30 min. After that, the membrane was soaked overnight in distilled water to separate it from the glass mold and dried at room temperature in air for 2 h. A thin layer (1 μm) of PTMSP was formed over the MF-4SC layer. The overall thickness of the composite was equal to 220 μm.

### 2.2. Current–Voltage Curves Measurements

Standard solutions of sodium chloride and hydrochloric acid were chosen as test substances for studying the properties of membranes, which made it possible to predictably change the properties of the membrane and evaluate the applicability of the proposed model for solutions of electrolytes of various nature.

The current–voltage curves, or CVCs, of the composite membrane MF-4SC/PTMSP were measured in a 0.05 M solution of HCl and NaCl, which was pumped at a volume rate of 15 mL/min, using the procedure described in [6,7]. A direct current was applied to the polarizing electrodes with a scanning rate of 1 × 10^−4^ A/s or 5 × 10^−5^ A/s using a Keithley 2420 SourceMeter (Keithley Instruments, Inc., Cleveland, OH, USA). The voltage drop, Δ*E*, across the test membrane was measured using Ag/AgCl electrodes connected to Luggin–Haber capillaries, which were installed on both sides of the membrane by the Keithley 2701 ethernet multimeter/data acquisition system (Keithley Instruments, Inc., Cleveland, OH, USA).

### 2.3. Contact Angles Measurements

Experiments were carried out to determine the contact angles during the spreading of water droplets and especially pure monoethylene glycol on both surfaces of the composite membrane MF-4SC/PTMSP using the Drop Shape Analyzer DSA100 (Kruss, Germany). The experiments were carried out on a dry membrane. The average value of the contact angle was determined based on three independent experiments for each side of the membrane and the liquid used. The error of measuring the contact angles was 0.1°.

### 2.4. Membrane Surface Investigation

The morphologies of the opposite bilayer membrane surfaces were investigated using atomic force microscopy (AFM) and scanning electron microscopy (SEM). Studies were carried out by the scanning probe microscope SmartSPM^®^-1000 (AIST-NT, Novato, CA, USA) in semi-contact mode using fpN11 cantilever (beam length—130 µm; hardness—2.6–9.8 N/m; resonance frequency—118–190 kHz; radius of curvature of the probe needles—10–25 nm) and by the scanning electron microscope JSM-6490LV (JEOL Ltd., Tokyo, Japan) with a tungsten cathode and the ability to operate under low vacuum.

## 3. Theory

**Figure 1:**
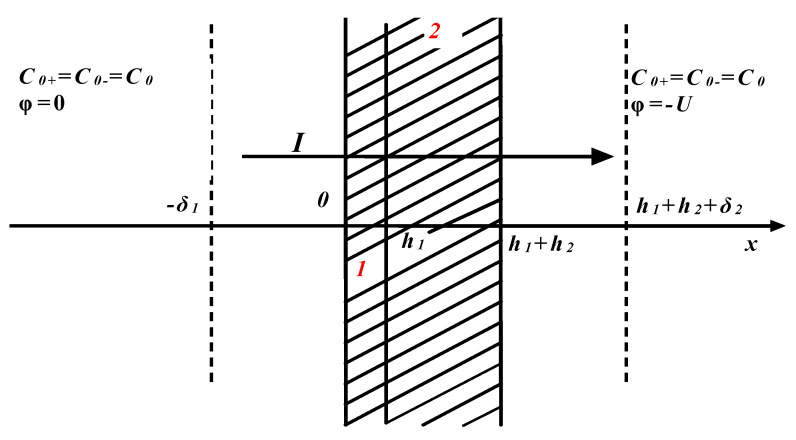
The scheme of electrodiffusion through bilayer membrane (s-orientation): 1-neutral layer; 2-charged layer; *C*_0_ = *C*_0±_—the equivalent concentration of 1:1 electrolyte in the intensively stirred areas of the cell; φ-electric potential.

The homogeneous model was earlier applied successfully to bilayer membranes consisting of the pristine MF-4SC layer and a layer modified by halloysite nanotubes, functionalized with polyaniline [7]. The homogeneous model of the fine porous bilayer membrane [1,3] is based on the Nernst–Planck approach, under electroneutrality conditions and equality of electrochemical potentials of electrolyte solutions at all interfaces. The membrane is characterized by the layer thicknesses *h*_1_ and *h*_2_ and diffusion coefficients *D* of an electrolyte molecule in the bulk solution and *D*_mi_ in the membrane layers (*i* = *1*, *2*), respectively; with the absolute value of charge densities of the fixed groups ρ_1,2_ *>* 0, i.e., exchange capacities, which are constant across each membrane layer, distribution coefficients γ_mi_ (*i* = *1*, *2*) of an electrolyte molecule inside the membrane layers, and thicknesses of the diffusion layers δ_1_ and δ_2_. Figure 1 shows a four-layer membrane system with the modified layer *1* orientation towards the flux of counterions (s-orientation). The reverse orientation (w-orientation) is occurred if the membrane in the measuring cell is faced by the main layer *2* also towards the flux of counterions (to the anode in this case, since the cation-exchange membrane is considered) or under change the direction of the external electric field. In [3], the boundary value problem, for the membrane system shown in Figure 1, was solved, and exact formulas were obtained for the full current–voltage curve for both orientations of a bilayer membrane in a cell filled with a 1:1 electrolyte solution and arbitrary constant exchange capacities, ρ_1,2_, of its cation layers.

In the case considered here, the first layer is assumed to be neutral (ρ_1_ = 0), so the formulas for the CVC are somewhat simplified, but they still remain difficult to use when processing experimental data, as follows:

(1) $$u=\left(\frac{i}{j}-1\right)\ln\frac{2+j\Delta_{2}}{2-j\left(\Delta_{1}+\frac{{\bar{\nu }}_{m1}}{1+H}\right)}+\ln\frac{\sqrt{{\bar{\sigma }}_{2}^{2}+{\left(2+j\Delta_{2}\right)}^{2}}-{\bar{\sigma }}_{2}}{\sqrt{{\bar{\sigma }}_{2}^{2}+{\left(2-j\left(\Delta_{1}+\frac{{\bar{\nu }}_{m1}}{1+H}\right)\right)}^{2}}-{\bar{\sigma }}_{2}}+\frac{\frac{j{\bar{\nu }}_{m2}H}{1+H}+\sqrt{{\bar{\sigma }}_{2}^{2}+{\left(2+j\Delta_{2}\right)}^{2}}-\sqrt{{\bar{\sigma }}_{2}^{2}+{\left(2-j\left(\Delta_{1}+\frac{{\bar{\nu }}_{m1}}{1+H}\right)\right)}^{2}}}{{\bar{\sigma }}_{2}}$$

(2) $$\frac{j\frac{{\bar{\nu }}_{m2}H}{1+H}+\sqrt{{\bar{\sigma }}_{2}^{2}+{\left(2+j\Delta_{2}\right)}^{2}}-\sqrt{{\bar{\sigma }}_{2}^{2}+{\left(2-j\left(\Delta_{1}+\frac{{\bar{\nu }}_{m1}}{1+H}\right)\right)}^{2}}}{{\bar{\sigma }}_{2}}=-\frac{i}{j}\ln\frac{\sqrt{{\bar{\sigma }}_{2}^{2}+{\left(2+j\Delta_{2}\right)}^{2}}-\frac{i}{j}{\bar{\sigma }}_{2}}{\sqrt{{\bar{\sigma }}_{2}^{2}+{\left(2-j\left(\Delta_{1}+\frac{{\bar{\nu }}_{m1}}{1+H}\right)\right)}^{2}}-\frac{i}{j}{\bar{\sigma }}_{2}}$$

where $i=\frac{I(h_{1}+h_{2})}{C_{0}FD}$ and $u=\frac{F}{RT}U$-dimensionless current density and voltage (*I* and *U* -dimensional current density and voltage, respectively); $H=\frac{h_{2}}{h_{1}}$, $\Delta_{1,2}=\frac{\delta_{1,2}}{h_{1}+h_{2}}$, ${\bar{\nu }}_{m1,m2}=\frac{D\gamma_{m1,m2}}{D_{m1,m2}}$, ${\bar{\sigma }}_{1,2}=\gamma_{m1,m2}\frac{\rho_{1,2}}{C_{0}}$; *j*-auxiliary parameter; *R*-gas constant; *T*-absolute temperature; *F*-Faraday’s constant.

Therefore, seeking the simplification, we will use the approximate current–voltage curve of the bilayer membrane with first neutral layer in case of an ideal (perm-selective) cation-exchange membrane (the transport number of counterions is equal to 1) [3], as follows:

(3) $$u=\frac{\sqrt{{\bar{\sigma }}_{2}^{2}+{\left(2+i\Delta_{2}\right)}^{2}}-\sqrt{{\bar{\sigma }}_{2}^{2}+{\left(2-i\left(\Delta_{1}+\frac{{\bar{\nu }}_{m1}}{1+H}\right)\right)}^{2}}+\frac{i{\bar{\nu }}_{m2}H}{1+H}}{{\bar{\sigma }}_{2}}+\ln\frac{\sqrt{{\bar{\sigma }}_{2}^{2}+{\left(2+i\Delta_{2}\right)}^{2}}-{\bar{\sigma }}_{2}}{\sqrt{{\bar{\sigma }}_{2}^{2}+{\left(2-i\left(\Delta_{1}+\frac{{\bar{\nu }}_{m1}}{1+H}\right)\right)}^{2}}-{\bar{\sigma }}_{2}}$$

In the same paper [3] it was shown that the approximate Formula (3) describes satisfactorily the results obtained by exact Formulas (1) and (2) when the asymmetry of the CVC is not too significant. On the other hand, when comparing strongly asymmetric CVC of different membranes, the error initiated by Formula (3) is systematic; therefore, these results can be compared with each other.

To estimate the magnitude of the limiting current densities at different orientations of the membrane, it is possible to use approximate formulas obtained in [6], when the following condition is met, $\frac{\rho_{2}}{C_{0}}\gg \frac{2h_{2}}{\delta }\frac{D}{D_{m2}}$, as follows:

(4) $$I_{\lim}^{s}=\frac{2FDC_{0}}{\left(\delta +h_{1}\gamma_{m1}D/D_{m1}\right)}+\frac{FD_{m2}}{h_{2}}\left(\sqrt{\rho_{2}^{2}+4\frac{C_{0}^{2}}{\gamma_{m2}^{2}}{\left(1+1/\left(1+\gamma_{m1}\frac{h_{1}}{\delta }\frac{D}{D_{m1}}\right)\right)}^{2}}-\rho_{2}\right)$$

(5) $$I_{\lim}^{w}=\frac{2FDC_{0}}{\delta }+\frac{FD_{m2}}{h_{2}}\left(\sqrt{\rho_{2}^{2}+4\frac{C_{0}^{2}}{\gamma_{m2}^{2}}{\left(2+\gamma_{m1}\frac{h_{1}}{\delta }\frac{D}{D_{m1}}\right)}^{2}}-\rho_{2}\right)$$

Formulas (4) and (5) are obtained under the condition of equal thicknesses of the diffusion layers, $\delta =\delta_{1}=\delta_{2}$, and consist of two terms, while the contribution of the first term, which is the density of the limiting current in the case of an ideally cation-exchange (perm-selective) membrane, is the main one. Its value is directly proportional to the diffusion coefficient of the electrolyte in the bulk solution and inversely proportional to the thickness of the diffusion layer. The first term in Formula (4) depends on the thickness of the neutral layer, the distribution coefficient in this layer, and ratio of the diffusion coefficient of the electrolyte molecule in the bulk solution to that in the neutral layer. The second term, which corrects for the non-ideality of the membrane, in both formulas, depends in a complex way on the concentration of the electrolyte, as well as the physicochemical and geometric parameters of both layers. The higher the exchange capacity of the charged layer, the smaller the contribution of the second term to the limiting current, all other parameters being equal. It can be seen from Formulas (4) and (5) that $I_{\lim}^{s}<I_{\lim}^{w}$, since both terms in Expression (4) are always less than the corresponding terms in Expression (5). Consequently, the density of the limiting current is higher when the charged membrane layer is facing the anode. In addition, it follows from Formula (4) that the neutral layer is a kind of additive to the diffusion layer. In this case, the limiting state at the orientation “s” occurs at the interface of the membrane layers, and not on the outer surface of the modified layer 1. The influence of the thickness of the diffusion layer δ manifests itself differently on the values of the limiting current density at different orientations of the membrane in the cell: its decrease leads to an increase in the first term in both expressions (4) and (5), but a decrease in the second term in (4), when the membrane is oriented by the modified layer 1 to the anode (s) and an increase in (5) when the unmodified layer 2 is oriented to the anode (w). As for the thickness, *h*_1_, of the neutral layer, its increase causes a drop in the density of the limiting current at the orientation “s” and an increase at the orientation “w”. That is, the asymmetry of the limiting current increases. An increase in the thickness, *h*_2_, of the charged layer leads, in both cases, to a decrease in the density of the limiting current. An increase in the diffusion coefficient, *D*_*m*1_, of electrolyte molecules in the first layer leads to an increase in $I_{\lim}^{s}$ and a fall of $I_{\lim}^{w}$, i.e., an increase in asymmetry, and an increase in the diffusion coefficient, *D*_m2_, of electrolyte molecules in the charged layer unambiguously leads to an increase in both values of the limiting current densities.

## 4. Results and Discussion

**Figure 2:**
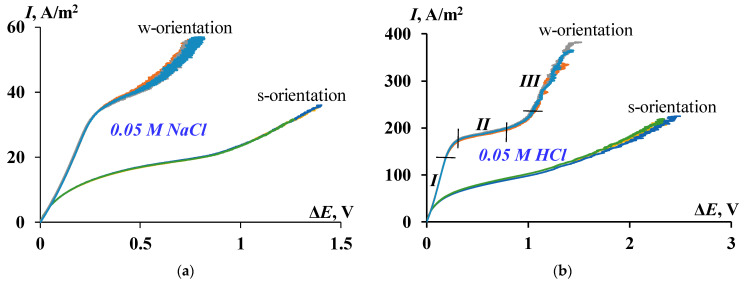
CVCs of bilayer MF-4SC/PTMSP membrane in 0.05 M NaCl (**a**) and HCl (**b**) electrolyte solutions. s-orientation-thin PTMSP layer is oriented towards anode; w-orientation-thick MF-4SC layer is oriented towards anode; I-ohmic region; II-limiting current plateau region; III-overlimiting region.

The experimental CVC of the cast bilayer sample of MF-4SC/PTMSP membrane has a typical form for ion-exchange membranes with three regions: the ohmic region, the limiting current plateau, and the overlimiting region (Figure 2).

Table 1 contains all major characteristics of the CVC. The ohmic region corresponds to the linear growth of current and voltage (Figure 2b, segment I). The counterion deficiency is gradually formed in the diffusion layer due to the current through the membrane is carried by only one kind of ions (counterions). When the limiting current is reached, the concentration of counterions drops to the minimum values and the electromembrane system falls into the limiting state. The formation of a limiting current plateau on the current–voltage curve is observed (Figure 2b, segment II). Furthermore, because of developing the coupled concentration polarization phenomena, an increase in the current density above its limiting value is observed, and a region of overlimiting currents appears on the current–voltage curve (Figure 2b, segment III).

The limiting current densities and the conductivities of the electromembrane system, which are defined as the angle of the ohmic part slope of the CVC (*dI/dE*_ohmic_), are expectedly higher in hydrochloric acid solution compared with sodium chloride solution for each orientation, which is explained by the high mobility of protons. The conductivity in the ohmic region in case of HCl electrolyte is six times higher than for NaCl solution and that in the region of overlimiting currents (*dI/dE*_overlim_), which is also higher for both orientations.

The conductivity, which is determined from the current–voltage curve, includes the conductivity of the membrane, the conductivity of two diffusion layers on either side of the membrane, and the conductivity of the solution layer between the Luggin–Haber capillaries and the diffusion layers. The difference in the conductivity of NaCl and HCl solutions is determined by the high mobility of protons, and the conductivity value of HCl solutions is always higher than the conductivity value of NaCl solutions. The membrane conductivity is also higher in the H^+^ form than the membrane conductivity in the Na^+^ form due to the higher mobility of H^+^ (9610 μm^2^/s) than Na^+^ (1350 μm^2^/s). It is obvious that the conductivity, determined from the current–voltage curve of the membrane, is always higher in the HCl solution than in the NaCl solution at any region of the current–voltage curve.

According to the proposed model, the bilayer membrane under consideration should have an asymmetric current–voltage curve. As can be seen from Figure 2 and Table 1, the substantial asymmetry of the current–voltage curves is registered, depending on which side of the membrane faces the counterion flux, that means different values of the limiting current densities and all other characteristics of the CVC. Thus, one can see, with the orientation “w”, that is, by the MF-4SC layer faced the counterion flux, the value of the limiting current density is 2.5 and 2.8 times higher in the case of NaCl and HCl solutions, respectively. The results are confirmed by the analysis of Equations (4) and (5), which demonstrates the lower limiting current density in the case of PTMSP layer is facing the anode (i.e., towards the counterion flux). The turning of the PTMSP layer to the anode leads to a decrease in the angle of the ohmic part slope of the CVC (*dI/dE*_ohmic_), i.e., the initial conductivity (at zero current) of the membrane decreases for both electrolytes. At the same time, the ratio of the conductivities for the “w” and “s” orientations in the ohmic region is approximately 1.8 times for both solutions, and in the overlimiting mode, the ratio is 3.8 times for an acid solution and 2 times for a sodium chloride solution. Even in comparison with the pristine MF-4SC membrane in HCl solution [7], the conductivity of the ohmic part of the bilayer membrane drops by more than 3 times when the perfluorinated layer is faced the anode. In addition, the extent of the limiting current plateau (Δ) also depends on the orientation of the sample to the counterions flux: a longer plateau is observed in the case of the “s” orientation, that is, by a layer of non-conducting polymer PTMSP faced the flux of counterions.

Basing on the contact angle measurements the average values of the contact angles are shown in Table 2. Therefore, we can conclude that the surface of the PTMSP layer is more hydrophobic than the surface of the MF-4SC layer, while the contact angles for monoethylene glycol (EG) differ slightly and characterize both surfaces as lyophilic to diatomic alcohol. Noticeable differences in the angles of wetting with water of the opposite surfaces of the bilayer membrane by more than 10° give us confidence that the thicknesses of the diffusion layers formed near these surfaces can vary in magnitude even with equal angular velocities of stirring the aqueous electrolyte solution.

Formulas (3)–(5) and the Mathematica-12 computational shell were used to calculate theoretical CVCs and compare them with experimental CVCs. Some of the physicochemical parameters of the MF-4SC layer (diffusion coefficients and coefficients of equilibrium distribution of electrolyte molecules in the layer, exchange capacity) were taken from our previous works [5,6,7]; the remaining parameters were determined by the procedure of fitting experimental and theoretical CVC curves to minimize the deviation between them. As for the neutral layer PTMSP of the membrane, its characteristics were unknown, so we assumed for certainty that there was no interaction of electrolyte ions with the matrix of this part of the membrane ($\gamma_{m1}=1$), and other parameters were also found by the fitting method. The calculation results are summarized in Table 3.

**Figure 3:**
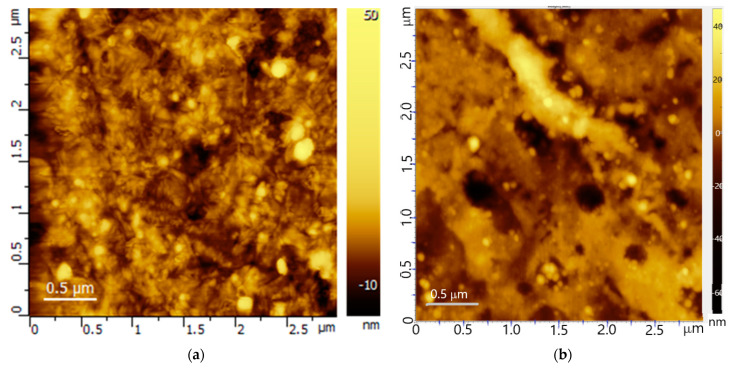
AFM images of the of the PTMSP surface (**a**) and the MF-4SC surface (**b**) of the bilayer composite membrane (3 µm × 3 µm) in semi-contact mode.

**Figure 4:**
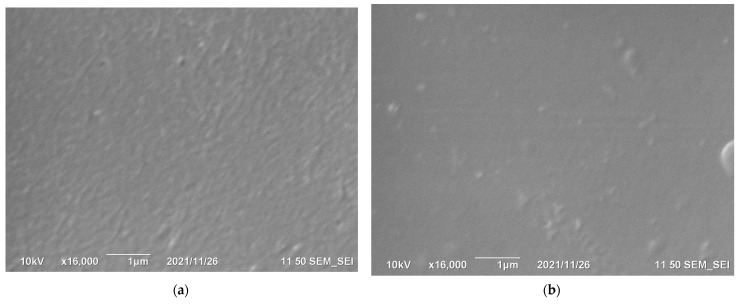
SEM images of the of the PTMSP surface (**a**) and the MF-4SC surface (**b**) of the bilayer composite membrane.

The last row in Table 3 represents the pristine membrane MF-4SC [7] for comparison. It can be seen that a bilayer membrane in a hydrochloric acid solution has a lower equilibrium distribution coefficient, which means that the positive sorption of ions in the pores of the membrane is higher than in the case of a sodium chloride solution. This coefficient in both layers is less than that of a single-layer MF-4SC membrane, which indirectly confirms the barrier properties of a thin neutral layer of PTMSP. The diffusion coefficients of electrolyte molecules in the neutral layer of PTMSP are about an order of magnitude lower than in the cation-exchange layer of MF-4SC. Moreover, the mobility of sodium chloride molecules in this layer is 5 times lower than that of hydrochloric acid molecules. In the MF-4SC layer, these mobilities differ by an order of magnitude, which quite qualitatively corresponds to the ratio of the mobilities of H^+^ and Na^+^ cations in a dilute electrolyte solution—9610 µm/s^2^: 1350 µm/s^2^ ≈ 7. The thickness of the diffusion layers on both sides of the membrane also differs by 10–20%, which indicates the influence of the degree of hydrophobicity of the membrane surface and its roughness on this value. Figure 3 show scans of both membrane surfaces obtained by atomic force microscopy. It can be seen that the surface of the PTMSP is more heterogeneous (Figure 3a) than the surface of the MF-4SC (Figure 3b), since the first was in contact with air during casting, and the second was in contact with polished glass. This circumstance could affect the thickness of the diffusion layers on different sides of the bilayer membrane. In addition, Figure 4a,b display scans of both membrane surfaces obtained by scanning electron microscopy, which obviously confirm the greater surface roughness of PTMSP (Figure 4a) as compared to MF-4SC (Figure 4b).

The pore size distribution of the PTMSP membrane is known from the literature [20,21,22]. The average pore radius is approximately 0.6–0.7 nm, i.e., it is sufficient for non-hydrated ions of the electrolyte solution and water molecules to pass through these pores. At the same time, the nanoporous membrane on the base of PTMSP has a barrier property in the aqueous ethanol mixture of 0–40 wt% of the ethanol content [23]. The threshold concentration of ethanol is between 40 and 50 wt% [23,24], depending on applied pressure differences of 10, 20, and 30 bar. A mathematical model of the onset of the water–alcohol mixture flow has been proposed in [24], and the percolation threshold, depending on the physicochemical and geometrical characteristics of the membrane system, has been found. The model suggests gradual membrane pore opening with an increase in pressure and alcohol concentration in the mixture and can take account of the distribution of alcohol molecules over the cross section of the membrane pore. In [24], we revealed that the effective microscopic contact angle in the pores of PTMSP membrane is obtuse and slightly decreases with an increase in the equilibrium concentration of ethanol, making the spherical shape of the meniscus closer to the planar one, that leads to hydrolyzation of the membrane pore walls. It is known that hydrophobic slippage significantly increases the critical pressure needed to push the fluid through the capillary [25]. Therefore, in our case, increasing ethanol concentration leads to better wetting of the walls of membrane pores and, consequently, to decreasing of the critical pressure drop (diminishing the percolation threshold). 

**Figure 5:**
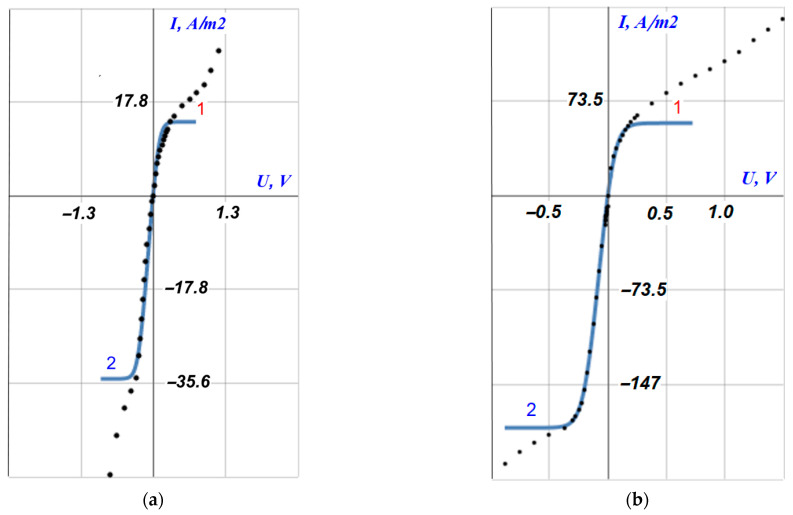
Asymmetrical current–voltage curves of bilayer composite membrane MF-4SC/PTMSP in 0.05 M solution of NaCl (**a**) and HCl (**b**): 1, s—orientation of the layer *1* (PTMSP) of the composite to the anode (towards the counter-ion flux); 2, w—orientation of the layer *2* (MF-4SC) of the composite to the anode; points—experiment; curves—theory.

Figure 5 shows a comparison of the theoretical total CVC (solid lines) of the MF-4SC/PTMSP bilayer membrane with experimental data (dots) for the case of 0.05 M solutions of NaCl (a) and HCl (b). There is a good coincidence of theoretical and experimental results up to the achievement of the limiting state by electromembrane systems. The mathematical model used here does not work in overlimiting current modes. Figure 5 indicates that the coincidence of theory and experiment is better for hydrochloric acid solution. At the same time, both the limiting currents density and the ohmic sections of the CVCs are described well enough for both electrolytes. Simultaneously, the addition of a thin neutral layer of a nanoporous PTMSP membrane reduces the specific conductivity of the bilayer membrane by more than 3 times, when its MF-4SC layer is oriented towards the anode, and by more than 5 times with the opposite orientation. The theoretical value of the specific initial conductivity of the membrane system (the tangent of the slope angle of the ohmic section of the CVC at the origin) can be found by differentiating the approximate Formula (3) for the CVC, as follows:

(6) $$\begin{array}{l} {\left(\frac{dI}{dE}\right)}_{\mathrm{ohmic}}={\frac{dI}{dU}|}_{I=0}=\frac{C_{0}F^{2}D}{RT(h_{1}+h_{2})}{\frac{di}{du}|}_{i=0}=\\ \frac{F^{2}}{RT}{\left(\frac{h_{2}}{D_{m2}\rho_{2}}+\frac{1}{2C_{0}}\left(\frac{\gamma_{m1}h_{1}}{D_{m1}}+\frac{\delta_{1}+\delta_{2}}{D}\right)\left(1+\sqrt{1+{\left(\frac{2C_{0}}{\rho_{2}\gamma_{m2}}\right)}^{2}}\right)\right)}^{-1} \end{array}$$

Note that the numerical value determined by Formula (6) should approximately correspond to the tangent of the slope angle of the ohmic section of the experimental current–voltage curve. Calculation by Formula (6), using data from Table 3 for HCl, gives a theoretical value ${\left(dI/dE\right)}_{\mathrm{ohmic}}=670\;\;{S/m}^{2}$, approximately coinciding with the arithmetic mean of the slopes of the ohmic section of the positive and negative branches of the CVC, respectively, equal to 755 and 432 S/m^2^ (Table 2). To find the initial electrical conductivity of a bilayer membrane, it is necessary to multiply (6) by its thickness, as follows:

(7) $$\kappa_{0}=(h_{1}+h_{2}){\left(dI/dE\right)}_{\mathrm{ohmic}}=\frac{F^{2}}{RT}\frac{h_{1}+h_{2}}{\frac{h_{2}}{D_{m2}\rho_{2}}+\frac{1}{2C_{0}}\left(\frac{\gamma_{m1}h_{1}}{D_{m1}}+\frac{\delta_{1}+\delta_{2}}{D}\right)\left(1+\sqrt{1+{\left(\frac{2C_{0}}{\rho_{2}\gamma_{m2}}\right)}^{2}}\right)}$$

For the HCl electrolyte from (7), we obtain κ_0_ = 0.15 s/m, while for the pristine membrane MF-4SC, this value, measured by the mercury contact method at alternating current, is 1.8 S/m [7], i.e., 12 times higher.

Using Formula (3), it is also possible to obtain an expression for the differential conductivity of a bilayer membrane (excluding diffusion layers), as follows:

(8) $$\kappa =\left(h_{1}+h_{2}\right)\frac{dI}{dU}=\frac{C_{0}F^{2}D}{RT}\frac{di}{du}$$

**Figure 6:**
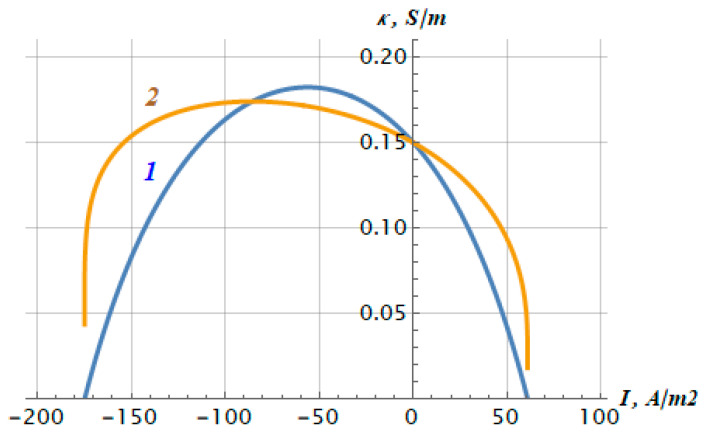
The dependence of the differential conductivity κ (curve 1) and the ratio (*h*_1_ + *h*_2_)*I/U* (curve 2) of the bilayer membrane MF-4SC/PTMSP in 0.05 M HCl solution, on the current density *I*.

The graph of the dependence of the electrical conductivity (7) on the current density is represented by curve 1 in Figure 6. For comparison, the same Figure 6 shows a graph of the dependence of the ratio (*h*_1_ + *h*_2_)*I/U* (curve *2*), which has a common intersection point with curve 1 at zero current.

**Figure 7:**
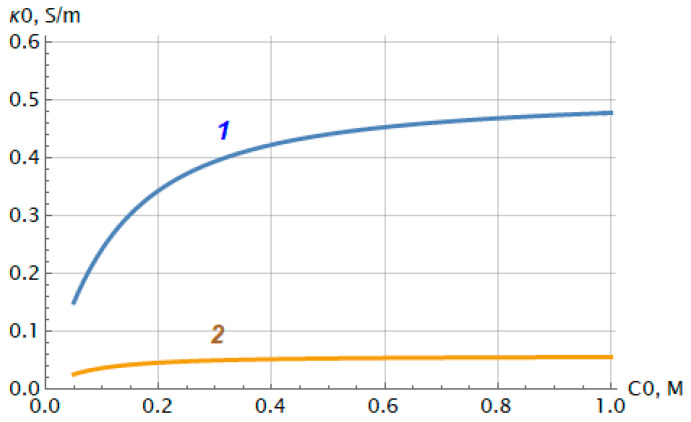
Predictive behavior of initial conductivity of bilayer membrane MF-4SC/PTMSP in HCl (curve 1) and NaCl (curve 2) solutions.

Using Formula (7), it is possible to predict the change in the initial electrical conductivity of the bilayer membrane (excluding diffusion layers) depending on the electrolyte concentration. Figure 7 shows this dependence in case of HCl (1) and NaCl (2) solutions.

Figure 7 demonstrates that the initial conductivity of the bilayer membrane behaves similarly to that of one-layer ion-exchange membranes [26,27]—it increases with increasing electrolyte concentration and tends to a constant value, $\kappa_{0}^{\infty }$, as follows:

(9) $$\kappa_{0}^{\infty }=\frac{F^{2}}{RT}\rho_{2}\frac{h_{1}+h_{2}}{\frac{h_{2}}{D_{m2}}+\frac{1}{\gamma_{m2}}\left(\frac{\gamma_{m1}h_{1}}{D_{m1}}+\frac{\delta_{1}+\delta_{2}}{D}\right)}$$

which is proportional to the exchange capacity of the charged layer.

## 5. Conclusions

Bilayer membranes, consisting of a thick layer of a pure perfluorinated membrane MF-4SC (Russian equivalent of Nafion^®^-117) and a thinner layer (1 μm) of the membrane, on a base of PTMSP, were prepared and investigated. Using the physicochemical characteristics of one-layer membranes MF-4SC and PTMSP, the asymmetric current–voltage curves of the bilayer composite were described with good accuracy, up to the limiting regime start in 0.05 M HCl and NaCl solutions. In this paper, studies of modified perfluorinated membranes were carried out in solutions of hydrochloric acid and sodium chloride. This is because information about the electrochemical behavior of such membranes in proton form is necessary to assess the prospects of their use as proton-conducting membranes in fuel cells. The study of perfluorinated membranes in sodium chloride solutions allows us to evaluate their electrochemical properties in sodium form—which is important when using these materials to obtain chlorine and alkali from sodium chloride solution—in the process of electrodialysis of multicomponent solutions, as well as detectors or sensors, since sodium chloride forms the mineral basis of natural waters, industrial solutions, and physiological fluids. This makes it possible to synthesize composite bilayer membranes based on MF-4SC and PTMSP, having significant asymmetry of CVC for using in electromembrane devices, such as membrane sensors, detectors, and diodes.

## Figures and Tables

**Table 1:** The characteristics of the CVCs of the bilayer and pristine membranes.

Membrane, Electrolyte, Orintation	*I*_lim_, A/m^2^	*I*_overlim_, A/m^2^	Δ*E*_lim_, V	*dI*/*dE*_ohmic_, S/m^2^	*dI*/*dE*_plateau_, S/m^2^	*dI*/*dE*_overlim_, S/m^2^	Δ*E*_overlim_, V	Δ, V
Bilayer, HCl, w-oriented	172	208	0.24	755	50	364	0.96	0.72
Bilayer, HCl, s-oriented	61	119	0.13	432	47	96	1.37	1.24
Bilayer, NaCl, w-oriented	35	43	0.28	129	28	64	0.57	0.30
Bilayer, NaCl, s-oriented	14	21	0.18	71	10	31	0.93	0.75
MF-4SC, HCl, h = 178 μm [7]	187.5	197	0.073	2390	16	292	0.65	0.57

**Table 2:** The contact angles on the PTMSP and MF-4SC surfaces of the membrane.

Polymer	θ, H_2_O	θ, EG
PTMSP	105°	73°
MF-4SC	93°	74°

**Table 3:** Theoretically calculated parameters for both layers of the bilayer, and pristine membrane.

Membrane, Electrolyte	$\begin{array}{l} I_{\lim}^{s},\;\\ {A/m}^{2} \end{array}$	$\begin{array}{l} I_{\lim}^{w},\;\\ {A/m}^{2} \end{array}$	$\begin{array}{l} D_{m1},\\ {\mu m}^{2}/s \end{array}$	$\begin{array}{l} D_{m2},\\ {\mu m}^{2}/s \end{array}$	$\begin{array}{l} \delta_{1},\\ \;\mu m \end{array}$	$\begin{array}{l} \delta_{2},\\ \;\mu m \end{array}$	$\gamma_{m1}$	$\gamma_{m2}$	$\begin{array}{l} \rho_{2},\\ \;M \end{array}$
MF-4SC/PTMSP, HCl	61	172	10.1	170	200	185	1.0	0.477	1.08
MF-4SC/PTMSP, NaCl	14	35	2.2	16.1	380	450	1.0	0.527	1.08
Pristine MF-4SC, HCl [7]	-	187.5	-	42.9	300	300	-	0.715	0.81

## Data Availability

Not applicable.
